# Pragmatic weight estimation for dosing of a new paediatric treatment for schistosomiasis prevention and control in preschool-aged children: a feasibility study in Uganda and Côte d’Ivoire

**DOI:** 10.1186/s40249-026-01507-9

**Published:** 2026-10-12

**Authors:** Nora Monnier, Prudence Beinamaryo, Mike Wells, Agathe Akoa, Moses Aritnaitwe, Tanja Barth-Jaeggi, Fidèle K. Bassa, Jenny Burrill, Gaoussou Coulibaly, Nana R. Diakité, Norbert N. Dje, Alexise D. Gnahore, Jan Hattendorf, Eveline Hürlimann, René O. Kouamé, Alain C. Kouamin, Hilda Kyarisiima, Isabelle L. Lange, Suzanne Lobohon Lavry, Betty Nabatte, Stella Neema, Eliézer K. N’Goran, Hahyat R. N’Goran, Ulrich Ohouo, Ashley Preston, Laura F. Roth, Tra J Sehi Bi, Dianne Verhoeven, Andrea S. Winkler, Alain Toh, Peter Steinmann, Mamadou Ouattara

**Affiliations:** 1 Swiss Tropical and Public Health Institute, Allschwil, Switzerland; 2 University of Basel, Basel, Switzerland; 3 Vector Control Division, Ministry of Health Uganda, Kampala, Uganda; 4 Division of Emergency Medicine, University of the Witwatersrand, Johannesburg, South Africa; 5 Jackson Memorial Hospital, Miami, FL USA; 6 Programme National de Nutrition, Ministère de la Santé, de l’Hygiène et de la Couverture Maladie Universelle, Abidjan, Côte d’Ivoire; 7 UFR Biosciences, Université Félix Houphouët-Boigny, Abidjan, Côte d’Ivoire; 8 Centre Suisse de Recherches Scientifiques en Côte d’Ivoire, Abidjan, Côte d’Ivoire; 9 Unlimit Health, London, UK; 10 Programme National de Lutte Contre les Maladies Tropicales Négligées à Chimiothérapie Préventive, Ministère de la Santé, de l’Hygiène Publique et de la Couverture Maladie Universelle, Abidjan, Côte d’Ivoire; 11 Département de Sociologie, Université Félix Houphouët-Boigny, Abidjan, Côte d’Ivoire; 12 Department of Neurology, TUM University Hospital and Center for Global Health, School of Medicine and Health, Technical University of Munich, Munich, Germany; 13 Department of Infectious Disease Epidemiology and International Health, London School of Hygiene & Tropical Medicine, London, UK; 14 Department of Sociology and Anthropology, Makerere University, Kampala, Uganda; 15 Department of Community Medicine and Global Health, Institute of Health and Society, University of Oslo, Oslo, Norway; 16 Present Address: Swiss Tropical and Public Health Institute, Kreuzstrasse 2, CH-4123 Allschwil, Switzerland

**Keywords:** Arpraziquantel, Child health, Implementation research, Neglected tropical diseases, Pediatric drug dosing, Preschool-aged children, Schistosomiasis, Weight estimation

## Abstract

**Background:**

Schistosomiasis remains a major public health concern in endemic settings. The Pediatric Praziquantel Consortium has developed a new paediatric treatment, arpraziquantel, for preschool-aged children. Because the treatment is dosed according to body weight, dose determination by scales poses operational challenges in community-based mass treatment campaigns. This study assessed the performance of the Pediatric Advanced Weight Prediction in the Emergency Room Extra-Long Mid-Arm Circumference (PAWPER XL-MAC) tape for weight estimation during mass drug administration in Uganda and Côte d’Ivoire.

**Methods:**

A mixed-methods study evaluated feasibility in terms of accuracy, reliability and acceptability of the PAWPER XL-MAC tape across delivery platforms and users between March 2025 and March 2026. Accuracy metrics comprised (1) the mean relative difference and mean absolute relative difference between predicted (tape) and observed (scale) weight and (2) the proportion of estimates within 10% (PW10) and 20% (PW20) of observed weight. Reliability was evaluated by calculating the intraclass-correlation coefficient (ICC). User experiences were collected through observations, questionnaires, focus group discussions and in-depth interviews.

**Results:**

In Uganda (*n* = 3369), mean absolute differences were 11.0–11.1%; PW10 60.2–61.0%. In Côte d’Ivoire (*n* = 1249), accuracy was higher (6.8–7.7%; PW10 72.4–78.2%). Reliability was high between users and countries (ICCs ≥ 0.84). Sufficient time for practical training was essential to achieve solid performance. With continued use, an increase in confidence, efficiency, and accuracy was observed. Qualitative results indicated strong acceptability and practical advantages amongst users.

**Conclusions:**

This is the first study to demonstrate that the PAWPER XL-MAC tape offers reliable, accurate, and acceptable weight estimation for community-based interventions, supporting its use in routine child healthcare and public health programmes.

*Trial registration* ClinicalTrials.gov ID NCT06698510, date of registration: 21.11.2024.

**Graphical Abstract:**

**Figure:**
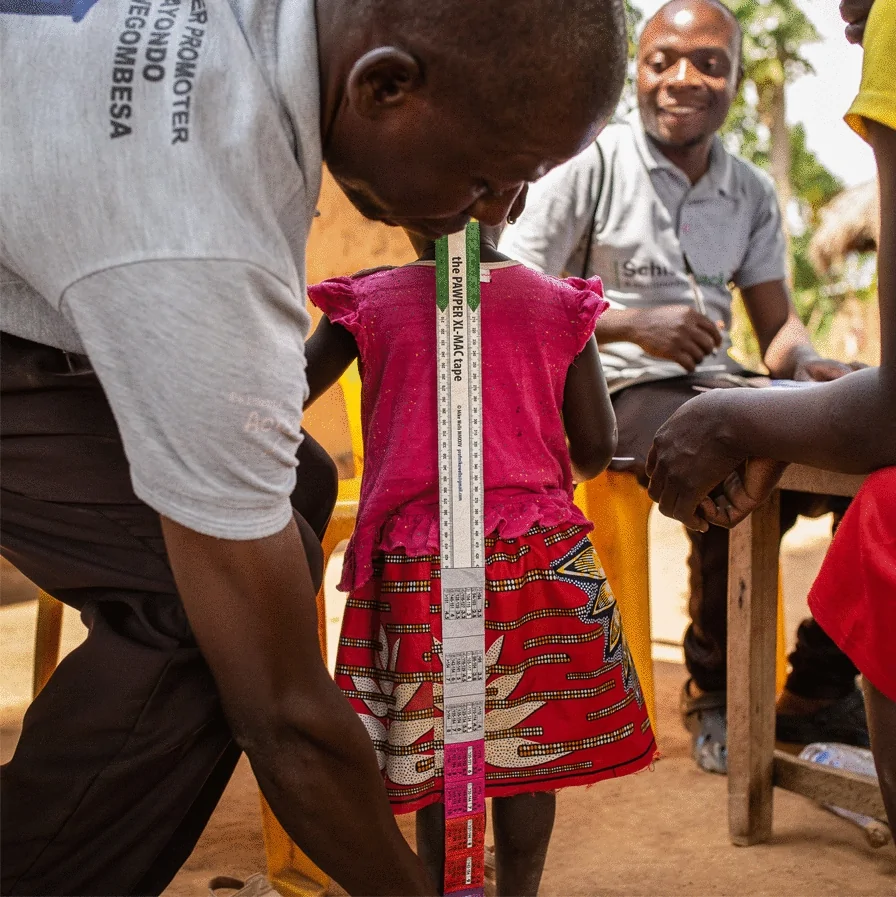

## Background

Schistosomiasis is a waterborne, parasitic neglected tropical disease (NTD) of major public health concern in many endemic areas, particularly sub-Saharan Africa. Preventive chemotherapy (PC) with praziquantel (PZQ) delivered through mass drug administration (MDA) campaigns is a successful public health intervention for morbidity control and prevalence reduction of schistosomiasis in endemic areas [1]. The multi-stakeholder Pediatric Praziquantel Consortium has developed a new dispersible paediatric treatment for preschool-aged children (PSAC) to address the challenges in mass treatment of PSAC related to the bitter taste and large tablet size of standard PZQ [2, 3]. The new formulation has received a positive scientific opinion from the European Medicines Agency (EMA) in December 2023 [4, 5]. Arpraziquantel (arPZQ) was included in the World Health Organization’s (WHO) List of Prequalified Medicines in May 2024 and in the WHO Essential Medicines List in September 2025 [6–8].

The Consortium was engaged in a rigorous implementation research programme, Adoption of Levo-Praziquantel 150 mg for Schistosomiasis in Endemic Countries—ADOPT, to generate evidence on the performance of different platforms and strategies for the delivery of a single dose of arPZQ to PSAC under programmatic conditions [9]. The overall aim of the ADOPT programme was to establish equitable access, enhance acceptance and maximise uptake of arPZQ in schistosome-endemic communities through validated implementation strategies and tools.

Similar to other PC-NTD drugs, arPZQ dosing is based on a defined weight range rather than a precise dose. The arPZQ dose is determined by body weight and based on a recommended target dose of 50 mg/kg for infections with *Schistosoma mansoni* and 60 mg/kg for infections with *S. haematobium* [3, 10]. Challenges associated with limited availability and operability of weighing scales in community settings call for pragmatic weight estimation methods to reliably and accurately determine body weight in the context of MDA in low-resource settings [11]. The aim of this study was to operationalize appropriate weight-based dosing under programmatic conditions. Our literature review and performance evaluation of existing paediatric weight estimation formulas and tools found higher and more consistent accuracy for length and habitus-based weight estimation methods such as the Pediatric Advanced Weight Prediction in the Emergency Room Extra-Long Mid-Arm Circumference (PAWPER XL-MAC) tape and Mercy tape, compared to one-dimensional methods based on age or height alone [11]. Height-based dose poles are used for dosing of azithromycin, ivermectin, and standard PZQ in MDA campaigns [12–14]. Two-dimensional weight estimation systems have been validated in paediatric clinical care settings and shown to be accurate across a wide variety of paediatric populations and geographies [15–18]. The additional measurement of the mid-upper arm circumference (MUAC) minimizes dosing errors resulting from over- or underestimation of body weight as it accounts for nutritional status which is particularly relevant in schistosomiasis-endemic settings where stunted growth or undernutrition are prevalent [19–21]. Based on our review and modelling, the two-dimensional PAWPER XL-MAC tape emerged as a potentially suitable, low-cost, pragmatic and broadly applicable method for weight estimation in resource-constrained programmatic and public health settings. Therefore, we investigated the feasibility expressed as accuracy, reliability and practical acceptability of the PAWPER XL-MAC tape as a novel weight estimation solution to derive recommendations for weight-based dosing of arPZQ in PSAC in community-based treatment settings and MDA during a small-scale pilot implementation in Uganda in 2025 and a subsequent large-scale distribution in Côte d’Ivoire in 2026.

We applied a mixed-method approach to assess feasibility related to the following objectives: (1) accuracy of the PAWPER XL-MAC tape for weight estimation, (2) the reliability of the weight estimation tape across different users, and (3) the practical acceptability, defined by acceptance, suitability and usability, based on observations, user feedback and experiences collected through questionnaires, in-depth interviews (IDIs) and focus group discussions (FGDs).

## Methods

### Study design and study sites

The PAWPER XL-MAC feasibility study was incorporated into the pilot implementation study for the distribution of arPZQ in Uganda and the large-scale distribution study in Côte d'Ivoire. In the former, the Integrated NTD MDA (INTD) programme, which delivers PC-NTD interventions to at-risk communities through a combination of fixed-point and door-to-door approaches using village health teams (VHTs) in endemic areas, was compared with the Integrated Child Health Days (ICHD) initiative. ICHD is an accelerated action initiative that promotes the health and wellbeing of children by offering biannual campaigns with intensified services that are delivered by health workers (HWs) at health facilities and by mobile teams at village outreach posts. Services include health promotion, deworming for children aged 1–14 years, and vitamin A supplementation for children aged 6–59 months. The initiative also provides a "mop-up" service for missed vaccinations. The two platforms investigated in Côte d’Ivoire were (i) the Programme National de Lutte contre Les Maladies Tropicales Négligées à Chimiothérapie Préventive (PNLMTN-CP), which contributes to reducing morbidity and mortality of endemic NTDs through PC, promotional, and research activities, and (ii) the Programme National de Nutrition (PNN), which carries out activities to promote and integrate nutrition into health services and community-based delivery of vitamin A supplementation for children aged 6–59 months. Both platforms rely on community health workers (CHWs) using door-to-door and fixed-point delivery for arPZQ treatment.

In Uganda, highly schistosome-endemic areas in Bugiri and Hoima districts were purposively selected for the PAWPER XL-MAC feasibility study. Along Lake Victoria in Bugiri district in eastern Uganda, the prevalence of infection with *S. mansoni* in PSAC is up to 35% [22]. In western Uganda along Lake Albert, the reported prevalence of *S. mansoni* infection in PSAC in Hoima district is 20% [23].

In Côte d’Ivoire, sites where a small-scale pilot study had been conducted in an earlier phase of the ADOPT programme in the Agboville and Bangolo health districts were retained for the current study. Agboville in southern Côte d'Ivoire is endemic for *S. mansoni* and *S. haematobium*, with the latter being more prevalent [24]. In western Côte d’Ivoire, the Bangolo district is considered highly endemic for *S. mansoni* [25, 26]. PC with arPZQ for PSAC alongside albendazole and standard PZQ for school-aged children was delivered through PNLMTN-CP in the Agboville health district where the PAWPER XL-MAC feasibility study was conducted in four villages, namely Grand-Morié, Rubino, M’Batra and Mafou. Arpraziquantel was distributed as part of the vitamin A supplementation campaign through the PNN in the Bangolo health district, where the feasibility study was conducted in the four villages of Bahibly, Bléniméouin, Bouobly and Goenie.

### Study procedures

Study participants, community volunteers, programme supervisors, district health officers and investigators were aware of the allocated platforms as masking or blinding was not applicable.

#### Training

Training for the PAWPER XL-MAC feasibility study was integrated into the training of VHTs, HWs and CHWs for the pilot and large-scale treatment distribution in Uganda and Côte d’Ivoire, respectively. Suitability and usability related to understanding of the concept and correct use of the novel weight estimation tape were observed and assessed during training and through a practical exercise. A questionnaire was pre-tested and used to collect trainees’ feedback as well as experiences with dose poles, scales and MUAC measurements. The questionnaires were regularly reviewed and assessed for completion.

#### Study population, weight estimation measurements and procedures

Aligned with routine programme activities, communities were sensitised and mobilised prior to and during treatment campaigns. PC with arPZQ was delivered through the selected platforms and offered to eligible PSAC aged 24–59 months following consent from their parents/caregivers. Children with acute or chronic illness, known allergy to PZQ, a history of seizures, as well as children who normally resided outside the study area were excluded from treatment.

**Fig. 1:**
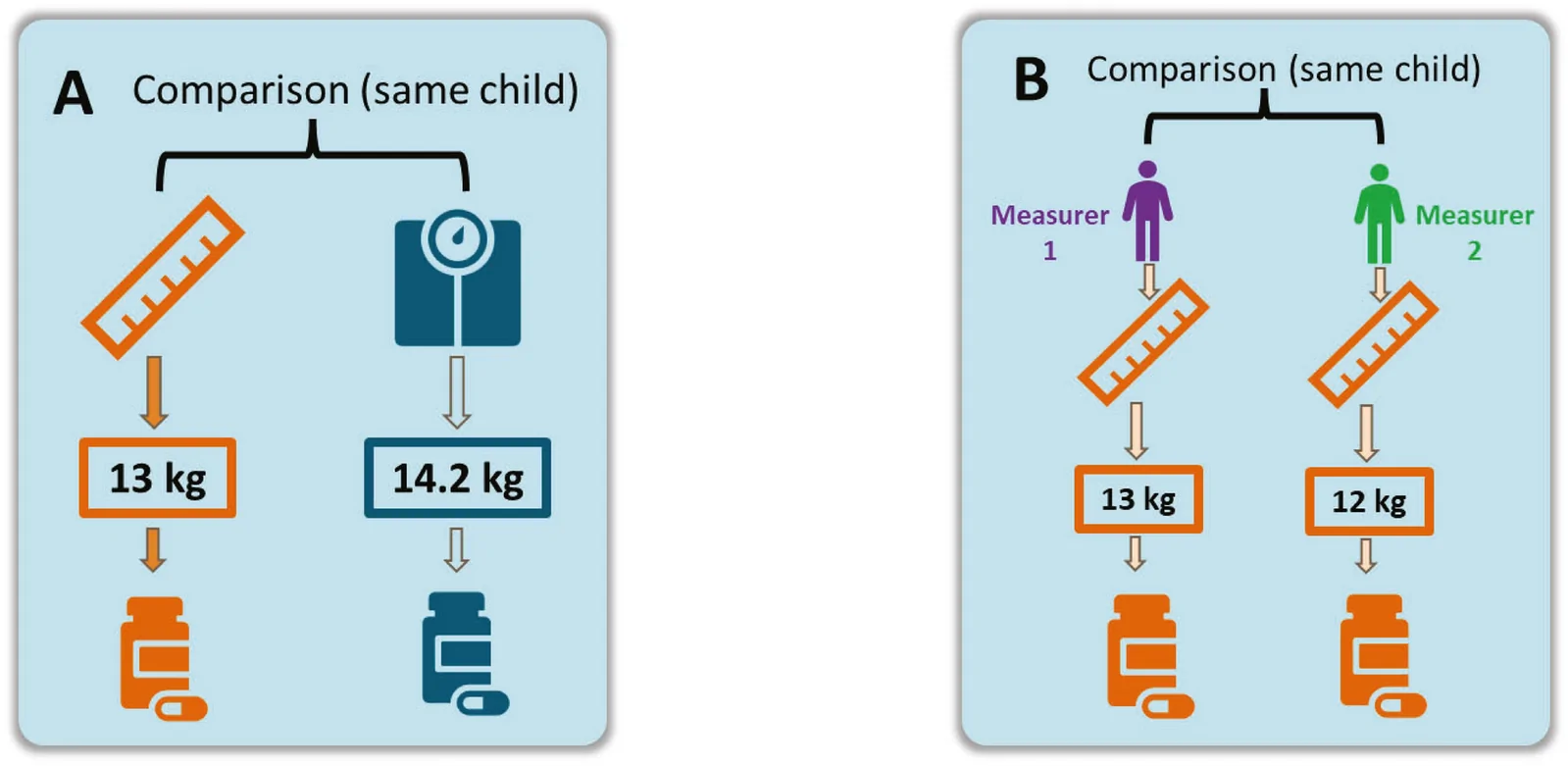
Measurement procedures for accuracy (**A**) and reliability (**B**): **A** Accuracy measured as difference in weight estimated by tape to scale-derived weight (reference weight). **B** Reliability evaluated by comparison of tape estimated weight according to two independent measurers

For the present study, weight estimations in kg were obtained by a designated measurer using the PAWPER XL-MAC tape on each child, which included measurement of the total body length followed by measurement of MUAC for weight adjustment. For accuracy, the tape-derived weight estimation and arPZQ dose were compared against the measured weight by mechanical (Uganda) or digital (Côte d’Ivoire) scales as shown in Fig. 1A. Every child in Côte d’Ivoire and every third child in Uganda was measured twice by different measurers (measurer 1 and measurer 2) to assess reliability (Fig. 1B). The final observed weight in each child was obtained with a weighing scale and used to determine the arPZQ dose actually administered, based on the target dose according to local endemicity or co-endemicity of the schistosome species [10].

#### Differences in weight measurement tools and procedures in Uganda and Côte d’Ivoire

**Fig. 2:**
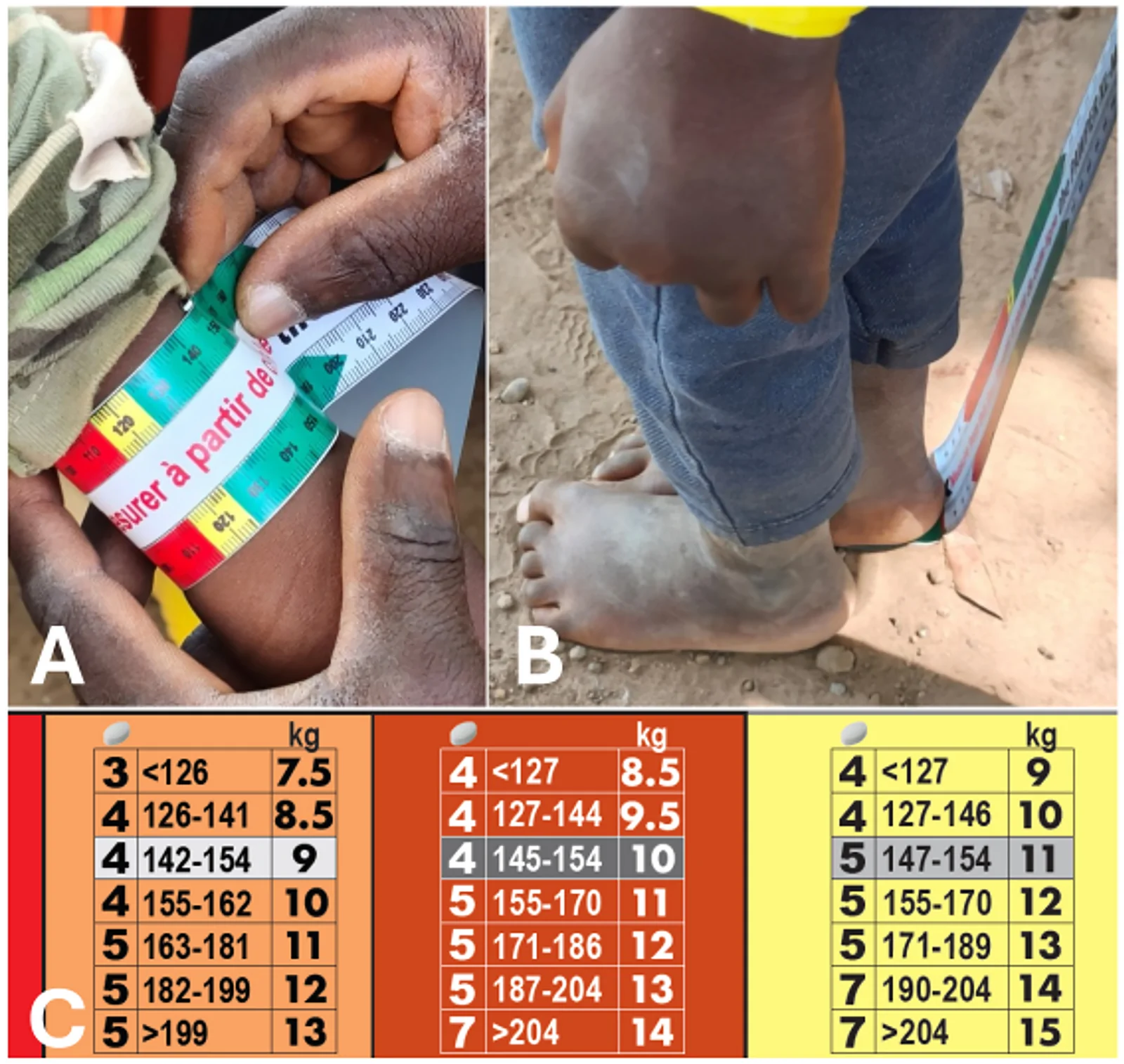
PAWPER MAC-XL tape adaptations for arpraziquantel distribution in Uganda and Côte d’Ivoire. **A**: MUAC malnutrition risk colour coding (applied in Uganda and Côte d’Ivoire); **B**: Foot extension for height measurement (in Côte d’Ivoire only); **C**: Integrated 60 mg/kg dose for arpraziquantel tablets (left column in each height compartment) (in Côte d'Ivoire only). *MUAC* mid-upper arm circumference in millimeters (middle column).

In Uganda, the original PAWPER XL-MAC tape (Tyvek material) was modified to support its use in a public health context by adding standardized colours (green, yellow, and red) related to the MUAC part and degree of malnutrition risk (Fig. 2A). The tape was printed one-sided for simplification of use. Tape length, colour scheme of length compartments, and original content were maintained. VHTs in INTD and HWs in ICHD performed the tape and scale weight measurements alongside procedures including registration of PSAC, drug preparation and (co)-administration of arPZQ, deworming, vitamin A supplementation and vaccination. Research assistants (RAs) supported and ensured adherence to the protocol. As reference for weight and final dose determination, mechanical weighing scales (model personal scale DT01, ZhongShan Jinli Electronic Weighing Equipment Co. Ltd., China) were used. In case the needle fell in between weights, the lower weight in full kilograms (kg) was noted as observed weight for dose determination.

Building on the experiences from the Ugandan pilot study, particularly the observed limitations of measurement accuracy related to the use of mechanical scales in Uganda, digital flat scales for mobile use (SECA 874, maximal weight 150 kg) with 100-g precision recommended by UNICEF were used in Côte d’Ivoire together with wooden planks to ensure stability on uneven ground. Furthermore, the PAWPER XL-MAC tape was modified by adding an extension on which to step to facilitate vertical height measurement (Fig. 2B). The arPZQ dose calculation was integrated into the tape (Fig. 2C). To mitigate errors in identification of height segments, each segment had a different colour. CHWs performed weight estimation across both platforms in Côte d’Ivoire, while the weight by scale for final arPZQ dose determination was obtained by trained programme assistants from either PNLMTN-CP or PNN, respectively. RAs supported the rotation and adherence to protocol and procedures, as in Uganda. The PAWPER XL-MAC feasibility study ended in Côte d’Ivoire once each measurer had achieved the required 30 measurements while in Uganda measurements were continued.

### Data collection and outcomes

All data related to demographic and professional background and experience of trainees, their feedback and observations from training were captured on paper-based forms. Similarly, all data related to tape measurements (MUAC, ideal and adjusted body weight in kg, arPZQ dose) were captured on paper-based forms. All data were entered into Excel for analysis. Observed scale-derived weight in kg and arPZQ dose were collected from paper-based treatment forms and double-entered into Excel for statistical analysis in R 4.5.1 (R Foundation for Statistical Computing, Vienna, Austria).

Accuracy of weight estimation based on the tape was compared with the observed weight by scale. For the analysis of the weight estimation performance, we used performance targets of PW10 > 75% and PW20 > 95%. The study outcomes in terms of accuracy were: (1) mean relative difference (bias) and mean absolute relative difference between the estimated weight (tape) and the observed weight (scale), and calculated as follows:

$$bias= \frac{Weigh{t}_{\left\{scale\right\}}- Weigh{t}_{\left\{tape\right\}}}{Weigh{t}_{\left\{scale\right\}}}$$

$$absolute \ difference= \frac{|Weigh{t}_{\left\{scale\right\}}- Weigh{t}_{\left\{tape\right\}}|}{Weigh{t}_{\left\{scale\right\}}}$$

(2) the percentage of tape weight estimates within 10% and 20% of the observed scale weight (PW10, PW20). Based on a systematic literature review by Wells et al., we adopted the proposed statistical performance targets for weight estimation systems, using 10% and 20% error margins as the primary benchmark metrics for assessing accuracy of estimated weight relative to a child’s actual weight. PW10 refers to the proportion of measurements not more than 10% above or below the true measurements (i.e. the weight estimate determined with the scale). Based on the recommendation, at least 70% of weight estimates should fall within 10% of the actual weight, while at least 95% should fall within 20% of the actual weight [15]. The accuracy was visualized using Bland and Altman plots. Inter-rater reliability was assessed using a two-way, single-measure intraclass-correlation coefficient (ICC) based on absolute agreement. We aimed for a sample size of 200 to assess inter-rater reliability. We refrained from a formal sample size estimation, because it is challenging to make assumptions about inter-rater reliability prior to data collection. The anticipated sample size of 200 was based on prior studies and expert opinion. In Uganda, a sample size of 200 was statistically sufficient to estimate reliability. Systematic random sampling was used to select children for the inter-rater reliability analysis for pragmatism. In Côte d’Ivoire, we measured every child to simplify procedures and protocols.

#### Social science data

In Uganda, the research teams conducted 10 IDIs with VHTs and HWs as well as four FGDs (two with VHTs and two with HWs), with participants purposively selected from across the distribution sites based on their use of the tape during arPZQ distribution. In Côte d’Ivoire, the social science team observed multiple instances of training and field use of the tape and conducted six IDIs and two FGDs with CHWs involved in the tape’s use. All FGDs and IDIs collected data on distributors’ experiences with the implementation of the arPZQ campaign, and asked specific questions for the weight-based dosing study, in particular their perceptions and experiences with the training of using the tape and scale, the tools’ application in the field, and their confidence in their reliability and ease of use. Non-participant observation focused on CHWs’ practices surrounding the learning and application of the tape in both training and distribution settings and offered the opportunity for informal chats about their experiences.

Trained RAs conducted interviews using semi-structured guides, audio-recorded them with consent, and translated (if necessary) and transcribed them verbatim. The team coded transcripts in MAXQDA using a combination of a priori and inductively derived themes (thematic analysis) and analysed the data through an iterative, team-based process to identify patterns in user experiences with the weight estimation tape across different contexts. The analysis explored users’ experiences with the PAWPER XL-MAC tape, including perceptions of its practical acceptability in terms of usability, acceptance and suitability in community settings. It also examined how these perceptions evolved over time, how the tape compared with existing tools, and how contextual factors shaped its use in practice.

## Results

**Fig. 3:**
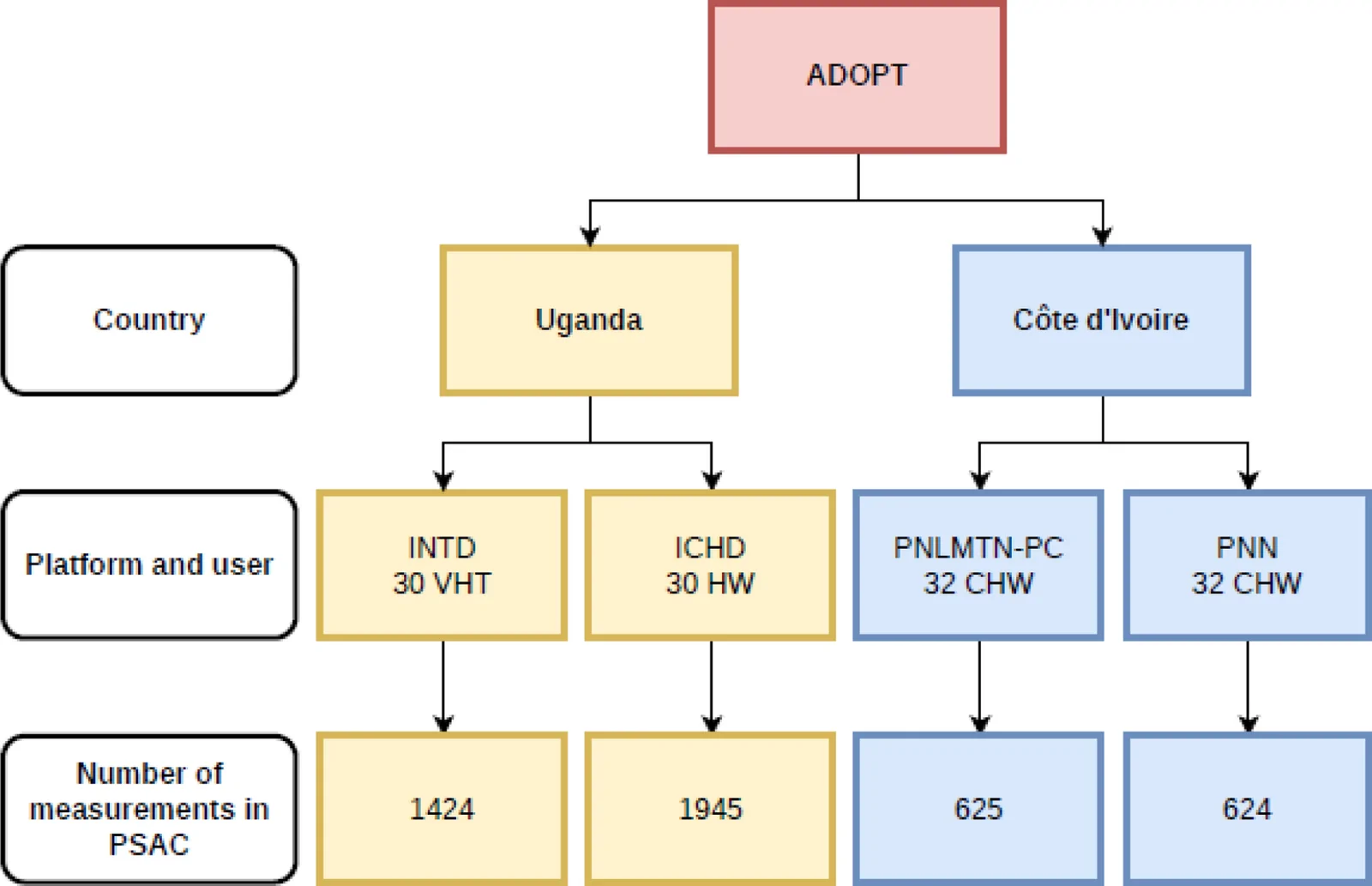
Flow chart describing the different users (VHTs, HWs, CHWs) of the PAWPER XL-MAC tape and number of measurements per country and platform (INTD, ICHD, PNLMTN-CP, PNN). *CHWs* community health workers*, HWs* health workers, *ICHD* Integrated Child Health Days, *INTD* Integrated Neglected Tropical Diseases, *PNLMTN-CP* Programme National de Lutte contre Les Maladies Tropicales Négligées à Chimiothérapie Préventive, *PNN* Programme National de Nutrition, *VHTs* village health teams

In Uganda, the PAWPER XL-MAC feasibility study was conducted during pilot distribution through INTD in March 2025 and through ICHD in May 2025, whereas in Côte d’Ivoire it was carried out in parallel by both platforms alongside large-scale distribution in January 2026. The number of tape-based measurements obtained by different users and included in the analysis is presented in Fig. 3. In Uganda, a total of 30 measurers per district were assigned to perform the tape measurements. In Côte d’Ivoire, 32 CHWs per platform were trained and performed the weight estimation by tape.

### Accuracy

**Fig. 4:**
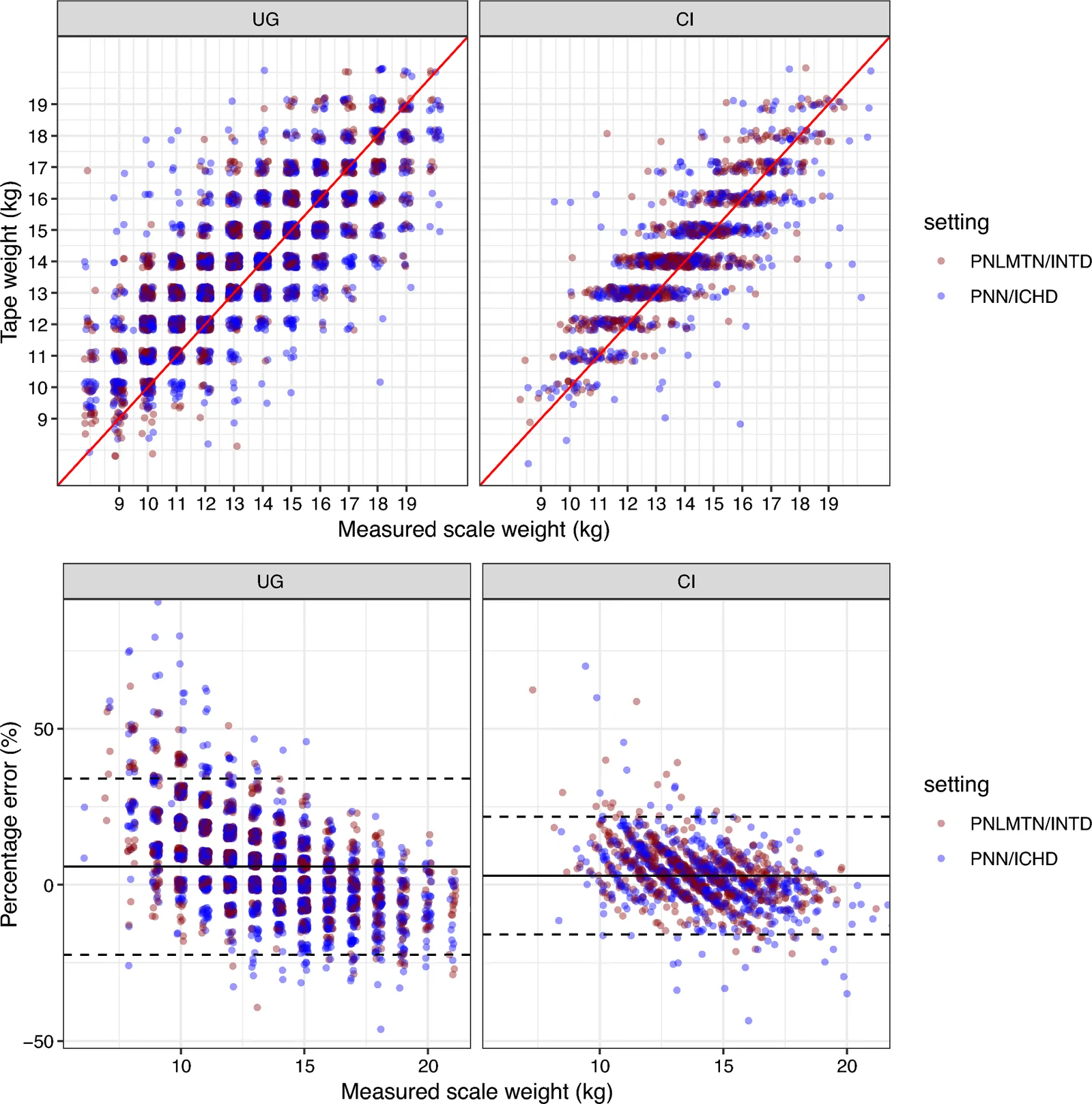
Scatter plots (top panels) and Bland Altman plots (bottom panels) showing discrepancies of scale and tape-based weight estimates. The solid line in the Bland Altman plots shows the mean difference between both methods. The dashed lines indicate 1.96 standard deviation above and below the mean difference. *CI* Côte d’Ivoire, *ICHD* Integrated Child Health Days, *INTD* Integrated Neglected Tropical Diseases, *PNLMTN* Programme National de Lutte contre Les Maladies Tropicales Négligées, *PNN* Programme National de Nutrition, *UG* Uganda

In Uganda, the bias of the tape measures was 5.9%, indicating that the tape systematically overestimated the weight (Table 1). The mean absolute difference between scale and tape was 11.1%. PW10 and PW20 were 60.7% and 86.3%, respectively. We found no noteworthy difference between the two platforms. Individual data points are presented in Fig. 4.

**Table 1:** Accuracy of tape-based weight estimation using scale-based weight as reference

Indicator	UG			CI		
	All	INTD	ICHD	all	PNN	PNLMTN-CP
Bias^a^	5.9	6.9	5.1	2.9	2.4	3.5
Difference^a^	11.1	11.1	11.0	7.2	7.7	6.8
PW10^b^	60.7	60.2	61.0	75.3	72.4	78.2
PW20^b^	86.3	85.7	86.8	95.6	94.7	96.5

All data are %, a Lower is better, b Higher is better. *CI* Côte d’Ivoire, *ICHD* Integrated Child Health Days, *INTD* Integrated Neglected Tropical Diseases, *PNLMTN* Programme National de Lutte contre Les Maladies Tropicales Négligées, *PNN* Programme National de Nutrition, *PW10, PW20* Proportion of tape weight estimates within 10%, 20% of observed scale weight, respectively, *UG* Uganda

In Côte d’Ivoire, the bias was 2.9% (Table 1) and the mean absolute difference was 7.2%. PW10 was 75.3% and PW20 was 95.6%, respectively. The PNLMTN-CP platform showed a slightly better performance.

### Reliability

**Fig. 5:**
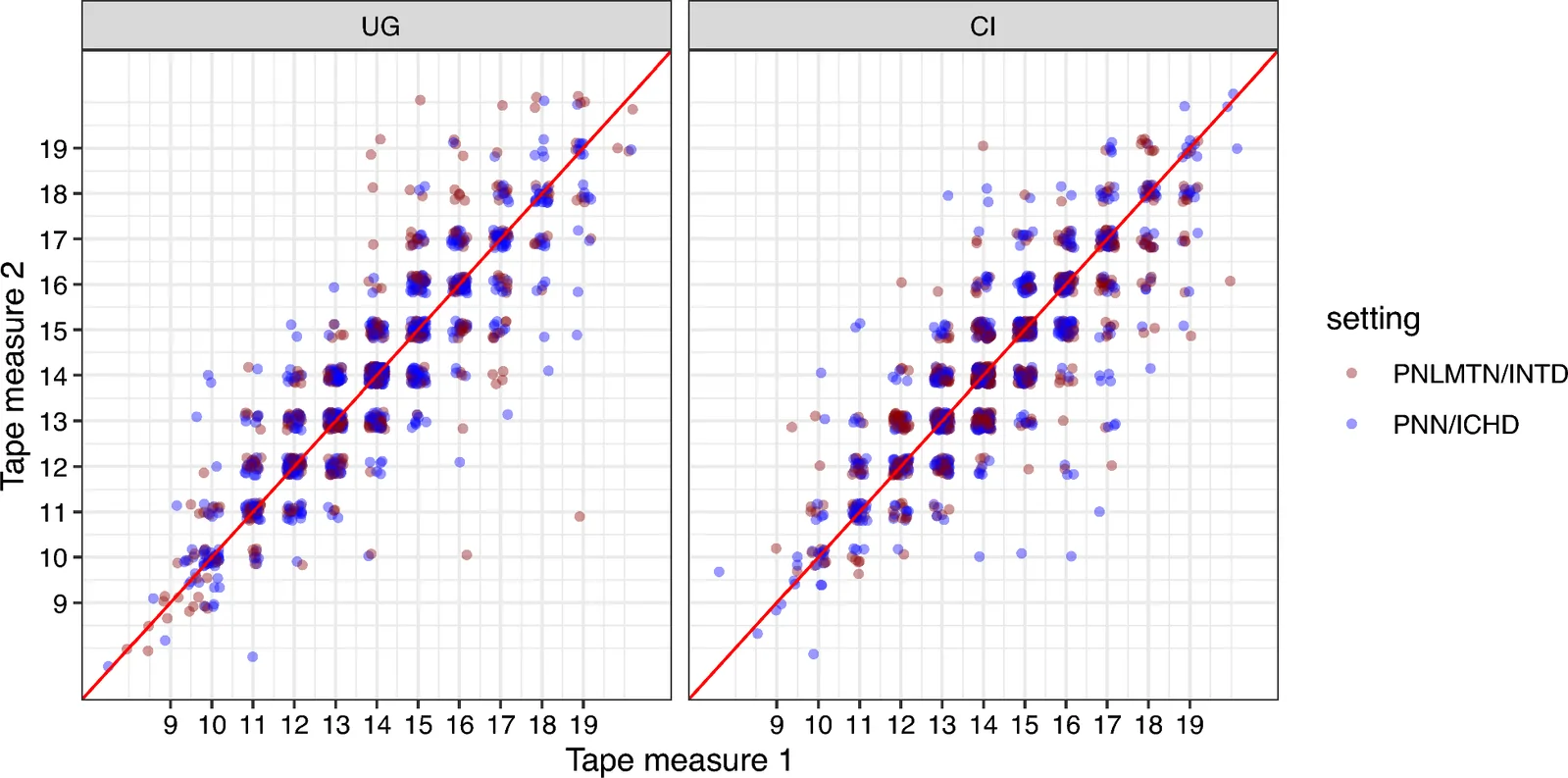
Consistency of tape performance across countries. *CI* Côte d’Ivoire, *ICHD* Integrated Child Health Days, *INTD* Integrated Neglected Tropical Diseases, *PNLMTN* Programme National de Lutte contre Les Maladies Tropicales Négligées, *PNN* Programme National de Nutrition, *UG* Uganda

Across platforms, similar consistency of the tape measures use was found in HWs in ICHD (ICC = 0.91) and VHTs in INTD (ICC = 0.89) in Uganda, and in CHWs in both PNLMTN-CP (ICC = 0.86) and PNN (ICC = 0.84) in Côte d’Ivoire, as shown in Fig. 5.

### Practical acceptability

Feedback and prior experience with the use of weight estimation tools (dose pole, scale and MUAC tape) and observations of the PAWPER XL-MAC tape during training and field use indicated its usability and suitability. During the practical exercise in Uganda in INTD, issues with incorrect sequence of measurements (9.1%), MUAC measurement (11.4%) and adjustment of weight based on MUAC (6.8%) were observed amongst VHTs. After resolution of issues, two VHTs were replaced due to a misunderstanding of concept or poor eyesight (4.5%). In ICHD, only one HW initially had issues with correct measurement of MUAC. It was noted that VHTs required more intensive practice because the use of both the scale and weight estimation tape was new to them in contrast to HWs who were familiar with the use of scales and MUAC tape. In Côte d’Ivoire, the practical exercise revealed several challenges, including initial difficulties positioning children correctly on the extension to obtain a straight, aligned height measurement and impaired MUAC measurement due to the extension (requiring folding or repeated wrapping to read the circumference). We also observed occasional difficulties among CHWs in correctly reading the measured values, and one CHW was replaced for this reason, while another was replaced due to a lack of understanding of the concept.

**Fig. 6:**
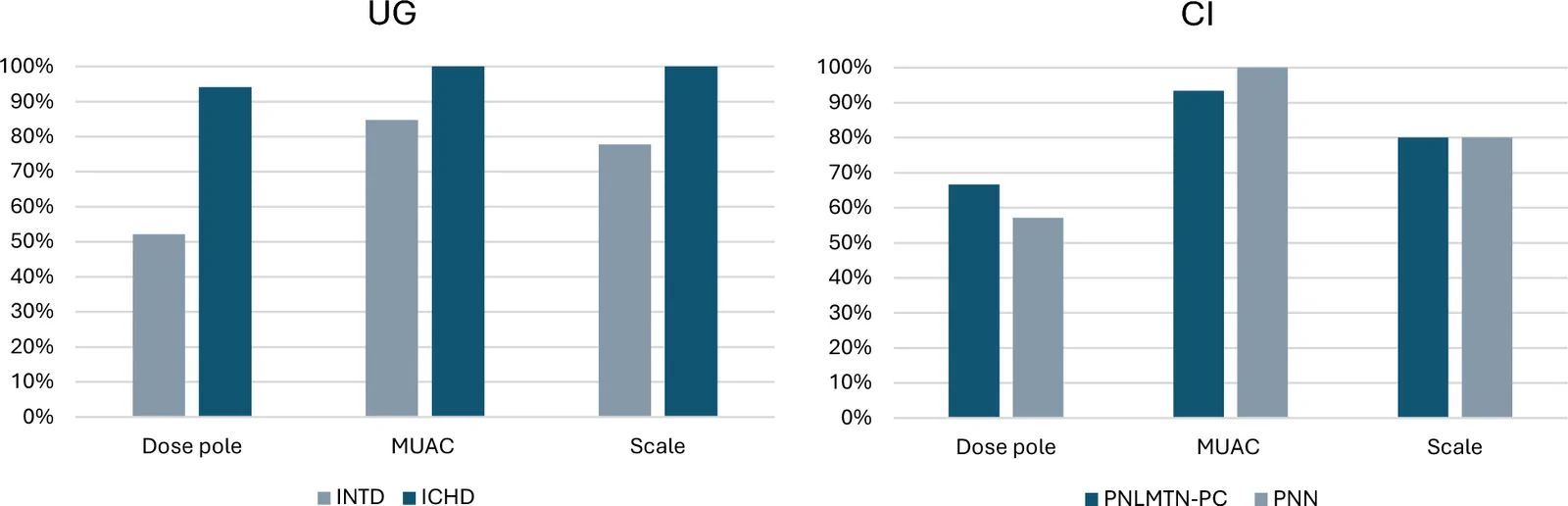
Self-reported experience of implementing staff with the use of dose poles, MUAC and scales in Uganda (*n* = 63) and Côte d’Ivoire (*n* = 29). *CI* Côte d’Ivoire, *INTD* Integrated Neglected Tropical Diseases, *ICHD* Integrated Child Health Days, *PNN* Programme National de Nutrition, *PNLMTN-CP* Programme National de Lutte contre Les Maladies Tropicales Négligées à Chimiothérapie Préventive, *MUAC* mid-upper arm circumference, *UG* Uganda

In Uganda, as shown in Fig. 6, only 52.2% of VHTs compared to 94.1% of HWs reported prior experience with the dose pole for PC-NTDs. As expected, all HWs had experience with MUAC tape and scale use compared with 84.8% of VHTs for the MUAC tape and 77.8% for the scale. In Côte d’Ivoire, in Agboville where PNLMTN-CP distributed drugs, 66.7% and 93.3% of CHWs had used the dose pole and MUAC tape, respectively, compared with 75.0% and 100% of CHWs involved in Bangolo, which was served by PNN. With regard to scales, 80.9% of CHWs in the PNLMTN-CP and 87.5% in the PNN had prior experience.

Based on self-reported feedback, eight VHTs and two HWs considered the time allocated for training and practice insufficient. All HWs and most VHTs (85–88%) found the PAWPER XL-MAC tape easy to learn and use. Only four VHTs and one HW reported difficulties, mainly related to printed numbers, dual measurements, and determining adjusted weight based on MUAC.

As in Uganda, training and practice time were frequently perceived as too short in Côte d’Ivoire. During one training of CHWs, some appeared to have difficulty distinguishing between similar colour shades of the tape, which occasionally seemed to contribute to errors in identifying the correct height segment. Informal discussions with programme supervisors and CHW supervisors suggested that some of the colour distinctions and labels used may not have aligned well with local ways of identifying colours. Very few participants (0.8%) reported challenges related to MUAC measurement, numbers, identifying adjusted weight, or double measurements. CHWs found the PAWPER XL-MAC tape easy to learn (87.7%) and use (90.0%).

Measurers across all sites reported similar initial challenges (position of tape at the heel, measurement of MUAC, adjustment of weight) when using the PAWPER XL-MAC tape. With corrections of errors under close supervision and continued use over time (training effect), both groups in Uganda and Côte d’Ivoire demonstrated and reported increasing confidence, efficiency and accuracy in applying the method. Health workers in Uganda generally appeared to grasp the technique more quickly than VHTs, reflecting their greater familiarity with anthropometric measurements, although both groups encountered similar practical difficulties during initial use. Most respondents reported that once the method was explained and practiced, it became easy to use.

“It is very easy. In the beginning it gave me a hard time after measuring the height, then the arm, adding up these to come up with kilograms first gave me a hard time. Being in green or any other colour to know the weight… it gave me some hard time for like two hours, but later I managed well.” (VHT Uganda)

Practical advantages of the PAWPER XL-MAC tape, particularly its suitability for MDA settings due to logistical benefits such as being lightweight and easy to carry, were highlighted by respondents in both IDIs and FGDs. Participants also perceived the PAWPER XL-MAC tape to provide accurate estimates of children’s weight, especially when compared with measurements obtained using a weighing scale. While some respondents initially expressed caution in adopting the tape, describing it as a new tool they needed to become familiar with, perceptions shifted with use. Both VHTs and HWs in Uganda and CHWs in Côte d’Ivoire expressed appreciation for the introduction of the weight estimation tape and viewed it as a useful and innovative tool for use in community settings.

“I think the tape is more practical for field use, especially in areas where scales might not be readily available or reliable.” (HW Uganda)

Despite these advantages, the tape was not universally perceived as straightforward or efficient in practice. Some respondents in Côte d’Ivoire described its use as relatively slow or cumbersome, particularly while they were still becoming familiar with the technique. Perceptions of its reliability were also shaped by how measurements compared with those obtained using weighing scales. Where discrepancies were observed, these could initially generate uncertainty about the estimated weight. Conversely, repeated comparison with scale measurements, together with supervision and practice, helped to establish confidence in the tape as a reliable dosing tool. Thus, confidence in the PAWPER XL-MAC tape developed not only through training but through its practical use and comparison with the established weighing method.

“Using a measuring tape in the field is more practical than using scales, especially when we have to move from one village to another or even from one neighborhood to another. Furthermore, handling scales is not easy because they can malfunction at any time” (CHW, CIV)

By combining qualitative and quantitative results, we identified a number of domains that are relevant for uptake of the PAWPER XL-MAC tape in public health interventions (Table 2).

**Table 2:** Summary of findings and program implications for weight-based dosing in community-based interventions

Domain/outcome	Key finding	Program implication
Accuracy	High accuracy of PAWPER XL-MAC tape for weight estimation	Consider dual-measurement methods for MDA & routine child health interventions & as alternative in absence of scales
Reliability & applicability	Reliable performance across users & countries, applicable across geographies & populations	Suitable for use across users & various healthcare & geographical settings & populations
Training, usability & capacity building	Easy to learn & use; performance improves with hands-on use	Emphasis on robust training, sufficient time for practice & refresher training
Acceptability & suitability	Highly accepted by VHTs, HWs, and CHWs; familiarity of measurements	Suitable for decentralized delivery platforms & task-shifting approaches
Operational advantages & costs	Lightweight, portable, durable Low-cost, reusable, locally printable	Recommended for MDA & resource-constrained settings Economically viable for national & repeated campaigns
Design considerations	Material (robust Tyvek) & layout (integrated dose, colours) for practical use & flexibility in measurement	Customization to program needs & local production (drug-specific tape, multi-purpose tool)
Additional value	Enables simultaneous malnutrition screening; alternative weight-based drug dosing	Multi-sectoral and program use, integration into routine child healthcare & decentralized delivery models

*CHWs* community health workers, *HWs* health workers, *MDA* mass drug administration, *PAWPER XL-MAC* Pediatric Advanced Weight Prediction in the Emergency Room Extra-Long Mid-Arm Circumference, *VHTs* village health teams

## Discussion

This is the first study evaluating the feasibility of using the PAWPER XL-MAC tape in a programmatic public health setting rather than a controlled clinical environment [27–29]. The lower accuracy of tape-based weight estimation observed in Uganda compared to Côte d’Ivoire was likely related to the lower precision of the mechanical reference scales (precision 1000 g) compared with the digital scales used in Côte d’Ivoire (precision 100 g), where the use of precise digital scales resulted in high accuracy of weight estimation that was comparable to that in previous studies assessing weight estimation methods in clinical settings [19, 30].

In contrast to the difference in methods and procedures for assessing accuracy of weight estimation, the comparison of tape reliability was not affected by the precision of the scales and demonstrated high reliability of measurements in both countries comparable to the findings from studies conducted in clinical settings [30].

The introduction of the PAWPER XL-MAC tape was generally well received across all sites and participant groups as a potentially useful tool for estimating children’s weight in the context of MDA and outreach. CHWs and HWs highlighted its practical advantages, particularly its logistical advantages and suitability for community-based distribution compared with heavier weighing scales. In Uganda and Côte d’Ivoire, the participants expressed strong appreciation for the tool and considered it innovative and helpful for community use. Familiarity with MUAC measurement among CHWs, VHTs, and HWs, together with experience using height-based dose poles for PC drug dosing supports its potential adoption in practice [12, 14, 31].

Our findings confirm and contribute to the evidence from our landscape analysis [11] and modelling study (unpublished manuscript) by demonstrating the tape’s performance in the context of MDA in low-resource settings. The PAWPER XL-MAC offers added value as a multipurpose tool for weight estimation, MUAC assessment, and nutrition screening, reducing the need for multiple tools and supporting integrated, decentralized service delivery and care models, particularly in resource-limited and decentralized settings. Its applicability across users, geographies, and population characteristics, combined with its familiarity and ease of use, may further support uptake among health workers and communities*.*

In summary, the triangulation of our mixed-method findings suggests that the PAWPER XL-MAC tape may serve as a practical complement or alternative to conventional weighing scales for determining arPZQ dose in community-based MDA and low-resource settings. Successful implementation, however, depends on robust training and initial supervision to ensure correct and confident use of the tool.

## Challenges and limitations

Training and distribution of the PAWPER XL-MAC tape occurred alongside pilot and large-scale programme activities of the ADOPT project rather than as a standalone study, creating operational constraints and placing a high information and task burden on CHWs and HWs. In Uganda, training coincided with programme activities related to the termination of USAID funding. This created overlapping demands on the workforce, as training and drug distribution coincided with malaria control activities. The need to recruit new VHTs during this period may have contributed to their more limited experience with the dose pole compared with HWs.

Across both countries, time constraints and an underestimation of the training required for tape use and proper weighing scale operation were observed, particularly among VHTs, who were generally less familiar with scales than HWs, for whom scale use and MUAC measurement are routine. Limited training time left trainees little opportunity for individual practice to gain adequate hands-on experience prior to distribution.

Direct comparison of weight estimation accuracy between Côte d’Ivoire and Uganda was not feasible due to methodological differences, including variations in the type and precision of reference weighing scales and country-specific tape modifications. The higher accuracy observed in Côte d’Ivoire may also reflect an improvement in measuring technique following supervised correction of initial measurement errors. Due to the potential influence from these corrections, we excluded the first ten measurements from the primary analysis. To account for potential bias, we conducted an additional sensitivity analysis (data not shown) including all measurements, also the first 10 in Côte d’Ivoire, which shows no noteworthy differences with respect to accuracy.

Operational challenges associated with different types of scales were observed across countries. The limited precision of mechanical scales and uneven surfaces requiring frequent calibration resulted in lower weight estimation accuracy in Uganda. Both mechanical and digital scales require a stable, flat surface for accurate measurement. Similarly, digital scales placed on soft but level surfaces produced lower weight readings compared with measurements taken on solid surfaces. Another limitation of this study is the assumption that the observed scale weight closely approximates the true weight, which may not necessarily be the case. Consequently, reference weights obtained from the scales used may not have represented true body weight, and the limited precision could influence comparisons with tape-estimated weights.

Several limitations related to the PAWPER XL-MAC tape were also identified. Correct use of the tape requires a basic level of literacy, numeracy, and adequate vision to read measurements accurately. Based on self-reported responses from CHWs and observations during training and distribution in Côte d’Ivoire, the foot or ‘step-on’ section of the tape was relatively long, which occasionally interfered with accurate MUAC measurement.

## Conclusions

The PAWPER XL-MAC tape is accurate, reliable, low-cost, and well accepted, making it a strong candidate for weight estimation in large-scale public health programmes, especially MDA campaigns and child health interventions in resource-limited settings. The tape design can be customized to reflect programmatic needs, thereby supporting efficiency and use by CHWs with minimal training.

## Data Availability

All related data have been presented within the article. The datasets supporting the conclusions of this article are available from the corresponding author upon request.

## Abbreviations

arPZQ: Arpraziquantel
CHW: Community health worker
CI: Côte d’Ivoire
FGD: Focus group discussion
HW: Health worker
ICC: Intraclass-correlation coefficient
ICHD: Integrated child health days
IDI: In-depth interviews
INTD: Integrated Neglected tropical diseases
MDA: Mass drug administration
MUAC: Mid-upper arm circumference
NTD: Neglected tropical diseases
PAWPER XL-MAC: Pediatric Advanced Weight Prediction in the Emergency Room Extra-Long Mid-Arm Circumference
PC: Preventive chemotherapy
PNLMTN-CP: Programme National de Lutte contre Les Maladies Tropicales Négligées à Chimiothérapie Préventive
PNN: Programme National de Nutrition
PSAC: Preschool-aged children
PW10, 20: Proportion of tape weight estimates within 10%, respectively 20% of observed scale weight
PZQ: Praziquantel
RA: Research assistant
UG: Uganda
WHO: World Health Organization
VHT: Village health team

## Declarations

### Patient and public involvement

Patients or the public were not involved in the design, or conduct, or reporting, or dissemination plans of our research.
